# Gift-giving intentions in pan-entertainment live streaming: Based on social exchange theory

**DOI:** 10.1371/journal.pone.0296908

**Published:** 2024-01-17

**Authors:** Zhi Zhang, Fang Liu

**Affiliations:** 1 School of Culture and Communication, Hebei University of Economics and Business, Shijiazhuang, China; 2 School of Literature and Journalism, Shandong University of Technology, Zibo, China; Sri Sivasubramaniya Nadar College of Engineering, INDIA

## Abstract

Pan-entertainment live streaming combines video with two-way communication and real-time viewer participation, allowing viewers to send virtual gifts to their favorite streamers. Drawing on social exchange theory, this study investigates the factors that influence viewers’ gift-giving intentions in live streaming from the perspectives of both viewers and streamers. It also explores the moderating role of streamers’ deceptive self-presentation. The theoretical framework is tested using AMOS and PROCESS Macro based on survey responses collected from 331 TikTok users in China. The results suggest that streamers’ attractiveness, expertise, parasocial interaction, and the viewers’ deceptive self-presentation significantly affect viewers’ gift-giving intention, and that streamers’ deceptive self-representation moderates the relationship between attractiveness, expertise, parasocial inter-action and the viewer’s consumer intention. These findings contribute to social exchange theory by highlighting the importance of streamers’ deceptive self-presentation in moderating the effects of attractiveness, expertise, parasocial interaction, and the viewer’s deceptive self-presentation on the viewer’s gift-giving intention.

## Introduction

In recent years, live streaming has emerged as a new social medium by combining video with two-way communication and real-time viewer participation [1]. This is primarily attributable to the rapid growth of the Internet and the widespread adoption of mobile devices. Social media users in China have flocked to live streaming in large numbers [2]. According to the 50th Statistical Report on Internet Development in China released by the China Internet Network Information Centre (CNNIC), as of June 2022, the user base for live streaming in China had reached 716 million, with an increase of 12.9 million users from December 2021 [3]. Therefore, live streaming platform research requires continuous attention. As a representative platform of China’s live broadcast platform, the original version of TikTok (one of the most widely used apps in China today) was a tool for users to produce, share, and watch short videos [4]. In China, viewers tune in for a wide range of pan-entertainment. While traditional live streaming primarily focuses on specific content such as gaming or personal vlogs, pan-entertainment live streaming encompasses a wider range of styles and forms, such as sports, variety shows, and concerts which have generated intellectual property rights related to online literature, movies, food, video games, and comics [5]. In a pan-entertainment live streaming show, streamers give showroom performances such as singing, dancing, musical instruments, and talk shows, e-commerce, personal knowledge sharing, and experience sharing [2, 6]. To obtain high traffic and user stickiness, pan-entertainment live streaming combines the benefits of the pan-entertainment industry with the characteristics of live streaming [7]. During pan-entertainment live streaming, viewers can interact with the streamer by sending texts, emojis, emoticon messages, and even presenting monetary virtual gifts to streamers [8]. Pan-entertainment live streamers generate their own virtual IP rights and earn income from viewers’ virtual gifts. As a result, the streamer’s image could influence viewers’ perception, trust, and purchase intentions [9]. According to Insight and Info (2021, March 29), viewers of China’s pan-entertainment livestreaming shows were predominantly male (nearly 60%), with those aged 24 and below constituting the highest proportion of the group (46.9%).

As gift-giving has become more prevalent in pan-entertainment live streaming, it has generated interest among scholars and researchers who are interested in understanding the motivations behind gift-giving behavior, the impact of gift-giving on streamers and viewers, and the broader social and cultural implications of this phenomenon [8, 10]. Previous studies have mainly concentrated on using “stimulus organism-response” model (SOR) [11], theory of planned behavior (TPB) [12], and technology acceptance model (TAM) [13] to understand how platforms are used to sell goods or services and attract tips from viewers through livestreaming. The identified factors for viewer tipping in live stream shows include viewer engagement [14], perceived utilitarian, hedonic, and symbolic value of live streaming shows [15, 16], live streamers’ characteristics such as attractiveness and source credibility [17–20], and social interactions [21–24]. Unlike viewers of e-commerce-focused live streaming whose primary motivations are linked to the products advertised, viewers of pan-entertainment live streaming shows purchase virtual gifts to foster romantic connections with streamers and to display a good image of other viewers [25, 26].

As a result, research into viewers’ viewing and gift-giving intentions must incorporate more nuanced variables. For instance, one of the critical aspects of pan-entertainment live streaming consumption is viewer’s sense of presence, which has received insufficient attention in the literature to date [27]. Self-presentation (i.e., how a viewer presents himself or herself online to shape how others observe him or her [28] and parasocial interactions (the one-sided relationship where a viewer extends emotional energy, interest, and time to live streamer, while the live streamer is unaware of the viewer [29] strengthen the connections between streamers and their viewers [30]. TikTok, as a famous live streaming app, has become ubiquitous, allowing users to establish an online persona that may or may not reflect their true identity [31].

In the context of online interactions, users may develop deceptive self-presentation, i.e., an individual’s intentional and strategic presentation of false or misleading information about himself or herself, especially in the context of online dating [31–37]. Meanwhile, in the context of pan-entertainment livestreaming, streamers who want to increase their attractiveness (thus maximize their revenue from live streaming) will engage in deceptive self-presentations during live streaming (such as using beauty cameras and creating a "perfect persona"). In contrast, viewers may also demonstrate deceptive self-presentation (online self-vanity) to highlight an identity status that is not present in reality (for instance, attracting the attention of the streamers by giving a large number of virtual gifts that do not match their economic strength), with some even aiming to form romantic relationships with streamers [38].

The need to close knowledge gaps and address practical problems in the pan-entertainment live streaming industry drives our investigation. Since there is limited studies that explore both the viewers’ efforts to send virtual gifts for the purpose of forming romantic connections and the streamers’ efforts to attract gift giving through deceptive self-presentation. In this unique context, this study aims to answer two research questions:

1. How do pan-entertainment streamers attractiveness and expertise, parasocial interaction, and viewers’ deceptive self-presentation collectively affect viewers’ gift-giving intention?
2. How does streamers’ deceptive self-presentation affect the above relationships?

Answering these research questions seems important as it helps unravel the dynamics between pan-entertainment streamers and viewers, especially regarding the antecedents of viewer behavioral intentions in pan-entertainment streaming. The results can help understand viewers’ psychological mechanisms, how they feel about the pseudo-social relationship with live streaming personalities, and how the different ways that viewers present themselves in livestream shows, thereby drawing a comprehensive view regarding their gift-giving intentions.

The value of this study lies in its examination of the intricate dynamics of pan-entertainment live streaming shows where viewers are motivated to create the desired two-way interactions with streamers through gift-giving. It not only investigates the factors influencing viewers’ intentions to give virtual gifts but also incorporates the crucial perspectives of both viewers and streamers’ deceptive self-presence. In particular, it examined the moderating effect of streamers’ deceptive self-presentation, providing a comprehensive understanding of the multifaceted nature of social interactions within the pan-entertainment live streaming environment. From a practical perspective, streamers and platforms may be able to improve their strategies with a better understanding of the factors that influence viewers’ gift-giving intention.

In order to inform the design of future live streaming platforms and services that support pan-entertainment live streaming, we provide a unique perspective on the opportunities and challenges of live streaming revealed by this study. In the present study, streamer traits such as attractiveness and expertise will be measured, along with deceptive self-presentation and para-social interaction, to determine their relationship to online gift-giving intention.

## Literature review

### Pan-entertainment live streaming in China

Pan live streaming services are based on social network sites, where each user can broadcast his or her program in real time via live streaming. Unlike other social media platforms, social live streaming services are synchronized, ensuring that all user activities occur simultaneously [39]. In other words, pan entertainment live streaming allows for real-time social interaction. Viewers can post questions and comments in real-time that are visible to the streamers [40], and the streamer can respond instantly. In addition to chatting with each other, live streaming viewers can communicate with the streamer in a variety of ways, including text chatting, team-based gaming, and gift-giving [41].

Because of its unique characteristics, live streaming has become increasingly prevalent all over the world. Due to the enormous amount of traffic, pan-entertainment live streaming in China has developed a new method of monetization: virtual gifts [22, 42]. In recent years, an ever-expanding repertoire of visual effects has served to bolster the perception of virtual gifting as a distinguished method of interaction between streamers and viewers [10]. Virtual gifts are more appealing than other forms of participation because of their visual cues and textual notifications; every virtual gift, from a free "star" to a ¥1,000 Lamborghini, is encoded with specially designed visual representations and textual messages [42].

### Social exchange theory

The social interactions between pan-entertainment live streaming viewers and streamers can be interpreted through the social exchange theory [43]. According to this theory, individuals engage in social interactions based on the rewards and costs associated with the interaction [44]. In the context of pan-entertainment live streaming, viewers may perceive streamers as social partners with whom they engage in an exchange relationship. Streamer may invest their attractiveness and expertise in their interactions with viewers who reciprocate through investments in virtual gifts. However, viewers may only be willing to reciprocate in this social exchange when they perceive it to be rewarding in terms of entertainment, social connection, and emotional fulfillment. This perception of benefits can then lead to a willingness to engage in gift giving activities. Giving gifts demonstrates a social exchange in this situation because viewers might send virtual gifts to streamers to gain recognition. This is particularly important for viewers who are engaged in parasocial interactions (i.e., one-directional interactions) hope to catch streamers’ attention.

In pan-entertainment live streaming shows, virtual gifts are displayed in the streaming room dashboards, once a viewer sends a virtual gift, the dashboard will display his or her name, and the amount of virtual money consumed [40]. This process allows viewers to enhance their social presence and establish a closer relationship with the streamer. In return, entertainment live streamers receiving virtual gifts may provide attention, recognition, and entertainment value to the viewers, creating a mutually beneficial exchange [45].

Overall, social exchange theory can provide insights regarding the motivations behind gift-giving on live streaming platforms and the role that gift-giving plays in shaping social relationships. However, previous studies have paid limited attention to the role of deceptive self-presentation from both the viewer and the streamers’ perspective. In the context of pan-entertainment livestreaming shows, both the viewers and the streamers may try to foster a false or unrealistic impressions themselves for the purpose of developing closer relationships and stimulating spending behaviours respectively. Therefore, integrating deceptive self-presentation could help understand the social and psychological processes underlying viewers’ gift-giving behavior in pan-entertainment livestreaming shows. The following section introduces the development of hypotheses that form the conceptual model of this study.

## Hypothesis development

### Attractiveness, expertise of streamer and viewers’ gift-giving intention

In this study, we define streamer attractiveness as the viewers’ perceptions of the streamer’s personality, appearance, and talent while live streaming [46]. Attractiveness is a determinant that can be recognized. Live streamers’ expertise in this study refers to their specialized knowledge, skills, and capabilities in creating and delivering live streaming content [47]. Prior studies have found that streamers have their own unique streaming aesthetic, area of expertise and attractiveness [20, 48] that influence viewers’ purchase intention. In the context of e-commerce live streaming, [49] have found that attractiveness and expertise of streamers are crucial factors on purchase intention. In pan-entertainment live streaming, the expertise of the streamer means talent and skill [40]. Personal attractiveness and expertise can arouse the appreciation of the viewer for the streamer, thereby generating consumption intention (gift-giving intention). Therefore, the following hypotheses are proposed:

H1: Attractiveness has a positive effect on gift-giving intention.
H2: Expertise has a positive effect on gift-giving intention.

### Parasocial interaction and viewers’ gift-giving intention

Para-social interaction is a simulacrum of conversational give and take in radio, television, and the movie, where the performer adjusts his/her performance to the expected response of the viewer [50]. This simulacrum of conversational give and take is called para-social interaction. Media and communication studies have been conducted to explore parasocial interaction relationships in a wide range of settings [22, 40, 51, 52]. In the context of pan-entertainment live streaming, particularly when the viewer size exceeds a certain threshold, the interaction between a streamer and a viewer may exhibit a unidirectional and one-to-many pattern [51]. Using the concept of "parasocial interaction," we attempt to describe the nature of interaction between a streamer and a viewer. Viewers believe that they are engaged in a face-to-face interaction and view media personas as "real friends" [52, 53]. This will increase personal attachment, relationship investment, and loyalty towards media figures [54].

In recent social media research, parasocial interaction was also employed to examine user behavior in relation to brand attitudes and purchase intentions within the context of e-commerce live streaming [11, 18, 19]. While some scholars have investigated the role of viewers’ text-based interaction with streamers [43] and emotional attachment to streamers due to expertise or physical attractiveness [8] in viewers’ gift giving. This is explained through parasocial interaction, which serves as an imagined friendship-like relationship, especially with streamers, which stems from an imagined intimacy or illusion [52] in which the viewer desires a romantic relationship with the streamer and is therefore willing to give gifts. Building on previous studies, this study proposes the following hypothesis to examine the impact of parasocial interaction as well as its interaction with deceptive self-presentation (H7). Thus, following hypothesis is proposed:

H3: Parasocial interaction has a positive effect on gift-giving intention.

### Viewer’s deceptive self-presentation and viewers’ gift-giving intention

Impression management is referred to as "self-presentation," and it refers to the way in which individuals work to influence the perceptions that others have of them [55]. In previous studies, deceptive self-presentation is prevalent and facilitated among online dating communities with the aim of developing romantic relationships with dating partners through deceptive self-presentation [31, 33–35, 39]. In the context of pan-entertainment live streaming, "deceptive self-deception" refers to the act of giving the anchor a valuable gift that does not match his economic status.

This study proposes that the motivation for this type of viewer to give gifts is comparable to the deceptive self-deception of online dating apps; that is, in order to build a perfect self-image and high social status in front of other viewers and streamers in face-to-face live streaming, they may aim to develop romantic relationships with the streamer, gain respect from other viewers, and highlight their social status by comparing with other viewers. Thus, following hypothesis is proposed:

H4: Viewer’s deceptive self-presentation has a positive effect on gift-giving intention.

### Streamer’s deceptive self-presentation as a moderator

Self-disclosure, the disclosure of personal information (i.e., thoughts or feelings) to others, is distinct from self-presentation [56]. Yet it is a crucial tool for those attempting to present their actual or ideal image [57]. Self-disclosure is a fundamental aspect of self-presentation, particularly online, where verbal disclosures are frequently highly controlled and audience-specific in the predominantly text-based social media environment [58]. Self-presentation refers to the process of communicating one’s desired social identity to others [59]. This study integrated self-disclosure and self-presentation to redefine the notion of a streamer’s deceptive self-presentation. According to a previous study [57], social interaction is ineffective if individuals do not present their identities. The presentation of a streamer lays the foundation for developing relationships between streamers and viewers in pan-entertainment live streaming. The negative streamer characteristics may affect online self-presentation [60]. In general, pan-entertainment streamers need to show their perfect persona during live streaming, such as ideal appearance, single status, and a way of expressing themselves that the viewers like.

Prior literature suggests that streamers’ self-presentation contributes to more effective purchase intention of virtual gifts [58]. Self-presentation promotes streamers’ expertise, attractiveness, and parasocial interaction to strengthen their intention to give gifts. On the other hand, under the deceptive self-presentation of the viewers, the deceptive self-presentation of streamers may make the viewers more willing to give gifts. As an essential feature, the deceptive self-presentation of streamers needs to be studied in terms of its moderating effects on the factors affecting gift-giving intention. Thus, following hypotheses are proposed:

H5: Streamer’s deceptive self-presentation moderates (strengthens) the relationship be-tween attractiveness and gift-giving intention.
H6: Streamer’s deceptive self-presentation moderates (strengthens) the relationship be-tween expertise and gift-giving intention.
H7: Streamer’s deceptive self-presentation moderates (strengthens) the relationship be-tween parasocial interaction and gift-giving intention.
H8: Streamer’s deceptive self-presentation moderates (strengthens) the relationship be-tween viewer’s deceptive self-presentation and gift-giving intention.

**Fig 1:**
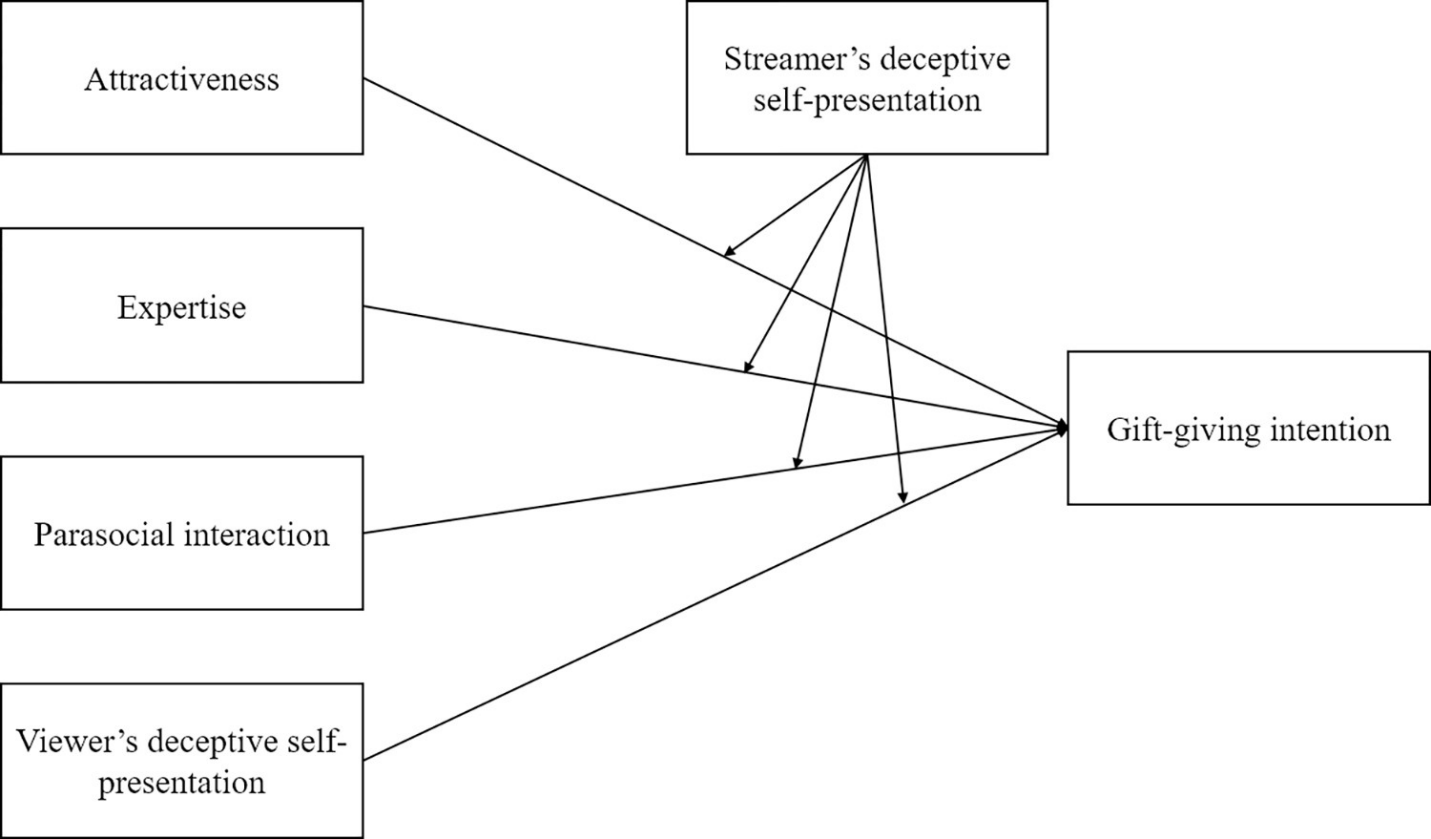
Conceptual model.

Conceptual model is presented as Fig 1.

## Materials and methods

### Data collection

After the survey was designed, we applied for the research ethics approval from the research committee of the School of Culture and Communication, Hebei University of Economics and Business (China), which reviewed and approved of the research design, methodology, and procedures involved in the data collection of this study.

After obtaining the research ethics approval, we gathered quantitative data to test our hypotheses using an online survey platform Wenjuanxing. Wenjuanxing is well recognized and utilized survey platform that allowed us to reach a diverse and representative sample of pan-entertainment live streaming viewers across China [61]. The respondents are pan-entertainment live streaming viewers on TikTok. To accurately examine the impact mechanism of live streaming gift-giving intentions, all respondents are live streaming platform consumers. On the survey webpage, we reassured respondents that they would remain anonymous throughout the survey and clarified the purpose of this study. While keeping the users anonymous, the survey targeted individuals who self-identified as pan-entertainment live streaming viewers by inviting them into a social media group. In the social media group, viewers were using pseudonyms and nicknames, thereby ensuring anonymity. Survey links were shared in the social media group.

With a lag time of two months between each survey, the data was collected using two waves of self-reported online surveys. [62] stated that this was done to lessen the possibility of common-method variance. In the first stage (March 4th to April 4th, 2023), we gathered information on attractiveness, expertise, par-asocial interaction, the viewer’s deceptive self-presentation, and the streamer’s deceptive presentation, while in the second stage (June 4th to July 4th, 2023), we gathered information on gift-giving intentions. The initial survey received 346 responses, while the subsequent survey received 331. The final sample consisted of 325 responses that were matched, which included respondents who responded to both surveys. Table 1 presents the result of demography information. 75.7% of respondents (N = 246) were male, while 24.3% were female (N = 79). This result concurs with the previous surveys regarding the dominant proportion of male viewers. Most respondents were between the ages of 19 and 40. The highest percentage of respondents with a 6100–8000 RMB income was 22.5% (N = 73). Regarding total spending on pan entertainment live streaming, respondents with a total expenditure of 1000 RMB or less accounted for the highest proportion, accounting for 26.2% (N = 85).

**Table 1:** Demography information.

Demography		Frequency	Percentage
**Gender**	Male	246	75.7%
	Female	79	24.3%
**Age**	19–25 years old	87	26.8%
	26–30 years old	88	27.1%
	31–35 years old	68	20.9%
	36–40 years old	67	20.6%
	41 years old and above	15	4.6%
**Income**	2000 RMB and below	70	21.5%
	2100–4000 RMB	54	16.6%
	4100–6000 RMB	61	18.8%
	6100–8000 RMB	73	22.5%
	8100 RMB and above	67	20.6%
**Total Spend**	1000 RMB and below	85	26.2%
	1100–2000 RMB	55	16.9%
	2100–3000 RMB	70	21.5%
	3100–4000 RMB	60	18.5%
	4100 RMB and above	55	16.9%

## Measures

All measures of this study were applied using 5-point Likert scales (1: Strongly disagree– 5: Strongly agree). A questionnaire was used to conduct an online survey and collect empirical data to examine the research model hypothesis. Because the survey was administered in China, we utilized the translation-back-translation method to ensure that the original English and Chinese instruments were consistent [63]. The items used to measure each construct (streamer attractiveness, expertise, parasocial interaction, viewer’s deceptive self-presentation, streamer’s deceptive self-presentation, gift-giving intention) were primarily derived from prior research. Some measures were modified slightly to accommodate live streaming.

The four items for measuring streamer attractiveness (ATR) were adapted from [46]. Expertise (EXP) was using a 3-item scale adapted from [64]. To capture parasocial interaction (PSI), we used a 3-item instrument developed by [65]. The six items for viewer’s deceptive self-presentation (VDSP) were modified from [66]. Gift-giving intention (INT) was used a 3-item scale adapted from [67]. Streamer’s deceptive self-presentation (SDSP) was 4-item scale adapted from [31]; based on the research content of this study, reverse scoring processing was performed in the analysis stage to achieve the measurement objective. All the items for measuring the constructs are attached and shown in S1 Appendix.

### Data analysis

As a self-reported questionnaire, this study used a time-lag method to collect data; however, there may be issues with common method bias. Consequently, Harman’s single-factor method is utilized to assess the severity of the common method bias [68]. AMOS 24.0 was used to examine the measurement model using confirmatory factor analysis (CFA). Following the procedure, we can confirm the measurement model’s validity, reliability, and conformity to the empirical data. The direct effects of the model’s hypotheses were examined by structural equation modelling analysis using AMOS 24.0. PROCESS Macro was used to test moderating effects. Following [69], simple slope plots of moderating effects were performed.

### Reliability and validity

To examine the measurement model’s adequacy, reliability and validity analyses were conducted. This study began by investigating the reliability and convergent validity. The fitting degree of the measurement model in this study is χ2/df = 1.163, CFI = 0.993, RMR = 0.039, TLI = 0.991, and RMSEA = 0.022, indicating that the structural validity of this study is good. Calculating the Cronbach alpha and composite reliability (CR) determined the instruments’ reliability. The Cronbach alpha and CR should be greater than 0.70. The assessment of convergent validity relies on examining the factor load and the average variance explained (AVE) [70]. Each item’s factor loading, and the AVE should be greater than 0.50. The model consisted of 23 items that described six latent constructs: streamer attractiveness, expertise, parasocial interaction, viewer’s deceptive self-presentation, streamer’s deceptive self-presentation, gift-giving intention.

Table 2 shows that the factor loadings on their respective constructs exceed the 0.7 thresholds. The composite reliability values and Cronbach alpha of the constructs all exceed the 0.7 thresholds. The AVE values all exceed the cutoff of 0.5. Therefore, the results indicate adequate convergent validity. The discriminant validity was then evaluated by calculating the square root of each AVE. The square root of each AVE must exceed inter-construct correlations [71]. Consequently, Tables 3 and 4 demonstrate that the discriminant validity of all proposed constructs can be guaranteed.

**Table 2:** Reliability and validity.

Construct	Item	Factor loading	Cronbach’s Alpha	CR	AVE
**Streamer Attractiveness**	ATR1	0.778	0.906	0.908	0.713
	ATR2	0.830			
	ATR3	0.815			
	ATR4	0.946			
**Expertise**	EXP1	0.806	0.800	0.801	0.573
	EXP2	0.730			
	EXP3	0.733			
**Parasocial interaction**	PSI1	0.696	0.826	0.831	0.622
	PSI2	0.865			
	PSI3	0.796			
**Viewer’s deceptive self-presentation**	VDSP1	0.791	0.929	0.930	0.688
	VDSP2	0.904			
	VDSP3	0.853			
	VDSP4	0.804			
	VDSP5	0.823			
	VDSP6	0.797			
**Streamer’s deceptive self-presentation**	SDSP1	0.766	0.883	0.884	0.656
	SDSP2	0.797			
	SDSP3	0.815			
	SDSP4	0.859			
**Gift-giving intention**	INT1	0.831	0.889	0.891	0.733
	INT2	0.797			
	INT3	0.934			

**Table 3:** Correlations and discriminant validity.

Construct	Mean	SD	1	2	3	4	5	6	7	8	9	10
**1.Gender**	1.243	0.430	-									
**2.Age**	2.492	1.216	-.159**	-								
**3.Income**	3.040	1.443	-0.016	.121*	-							
**4.TotalSpend**	2.831	1.433	0.077	0.043	0.064	-						
**5.ATR**	3.847	1.027	0.029	-0.064	-0.046	0.008	0.845					
**6.EXP**	3.766	1.012	0.029	-0.035	-0.013	-0.064	.453**	0.757				
**7.PSI**	3.621	0.848	0.014	-0.010	-0.015	-0.080	.483**	.427**	0.789			
**8.VDSP**	3.450	1.052	0.007	-0.018	-0.086	-.117*	.558**	.450**	.512**	0.830		
**9.SDSP**	3.681	1.027	-0.042	0.018	.120*	-0.013	.115*	0.016	.139*	.124*	0.810	
**10.INT**	3.716	1.022	0.001	-0.004	-0.072	-0.068	.457**	.431**	.461**	.493**	0.059	0.856

Note

**, p<0.01

*, p<0.05; ATR, streamer attractiveness; EXP, expertise; PSI, parasocial interaction; VDSP, viewer’s deceptive self-presentation; SDSP, streamer’s deceptive self-presentation; INT, gift-giving intention. The diagonal values are square roots of AVEs.

**Table 4:** Heterotrait-monotrait ratio (HTMT).

Construct	ATR	EXP	PSI	VDSP	SDSP	INT
**ATR**	-					
**EXP**	0.541***	-				
**PSI**	0.529***	0.514***	-			
**VDSP**	0.601***	0.517***	0.572***	-		
**SDSP**	0.130*	0.018	0.173**	0.144*	-	
**INT**	0.512***	0.513***	0.523***	0.536***	0.077	-

Note

***, p<0.001

**, p<0.01

*, p<0.05; ATR, streamer attractiveness; EXP, expertise; PSI, parasocial interaction; VDSP, viewer’s deceptive self-presentation; SDSP, streamer’s deceptive self-presentation; INT, gift-giving intention.

## Results

### Common method bias

Due to the use of self-reported cross-sectional data, Harman’s single-factor analysis was conducted to examine the potential for common method bias (CMB). The results indicate that the first factor accounts for only 37.982 percent of the variance, which is less than most of the covariance (less than 40%). Consequently, according to Harman, CMB is not a concern in this study.

### Hypothesis testing

**Fig 2:**
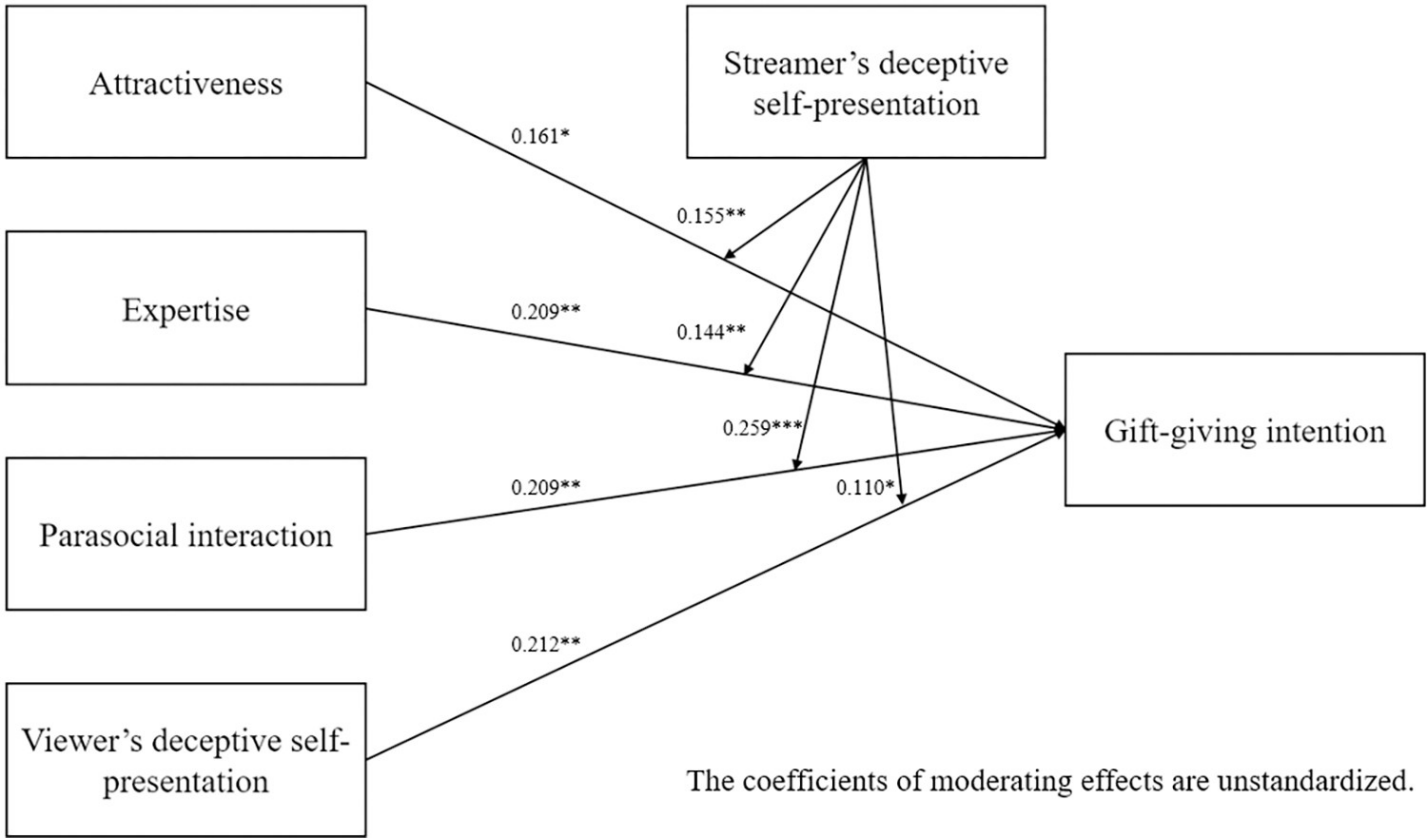
Results of model.

Direct effects of hypotheses testing were tested using AMOS 24.0. The results shown in Table 5 indicate that ATR (β = 0.161, p<0.05), EXP (β = 0.209, p<0.05), PSI (β = 0.209, p<0.05), and VDSP (β = 0.212, p<0.05) have significant positive effects on INT. Thus, hypotheses 1–4 are supported. The results of the model coefficients are illustrated in Fig 2.

**Table 5:** Results of direct effects.

Hypothesis	Path			STD.Estimate	S.E.	C.R.	P	Results
**H1**	INT	<—	ATR	0.161	0.075	2.317	0.021	Supported
**H2**	INT	<—	EXP	0.209	0.071	2.888	0.004	Supported
**H3**	INT	<—	PSI	0.209	0.111	2.890	0.004	Supported
**H4**	INT	<—	VDSP	0.212	0.068	2.992	0.003	Supported
Model fit: χ2/df = 1.273, RMR = 0.038, TLI = 0.989, CFI = 0.990, RMSEA = 0.029								

Note: ATR, streamer attractiveness; EXP, expertise; PSI, parasocial interaction; VDSP, viewer’s deceptive self-presentation; INT, gift-giving intention.

**Fig 3:**
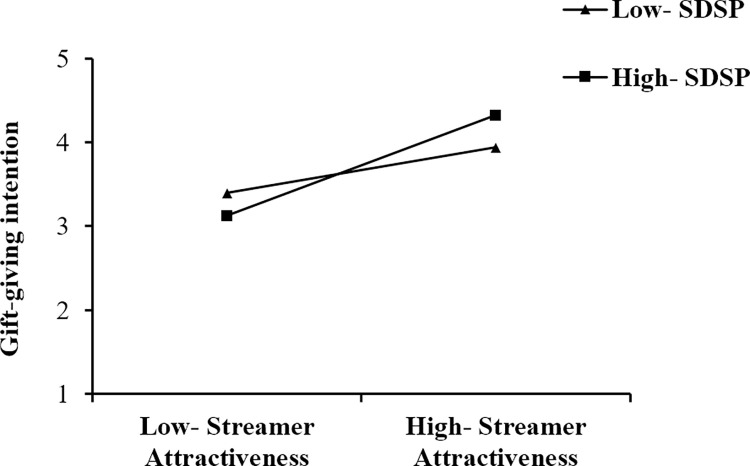
The moderating effect of SDSP on the relationship between ATR and INT.

**Fig 4:**
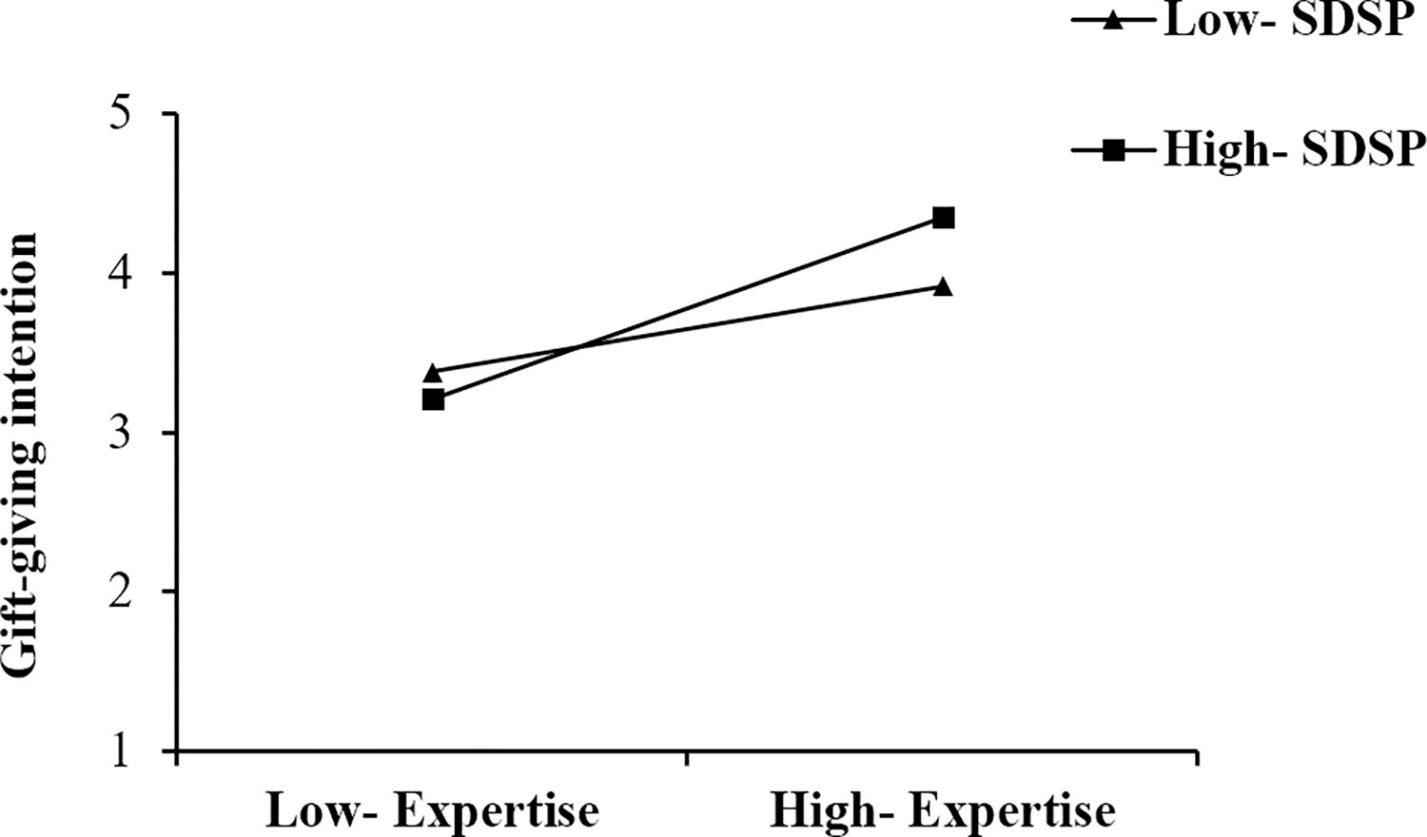
The moderating effect of SDSP on the relationship between EXP and INT.

**Fig 5:**
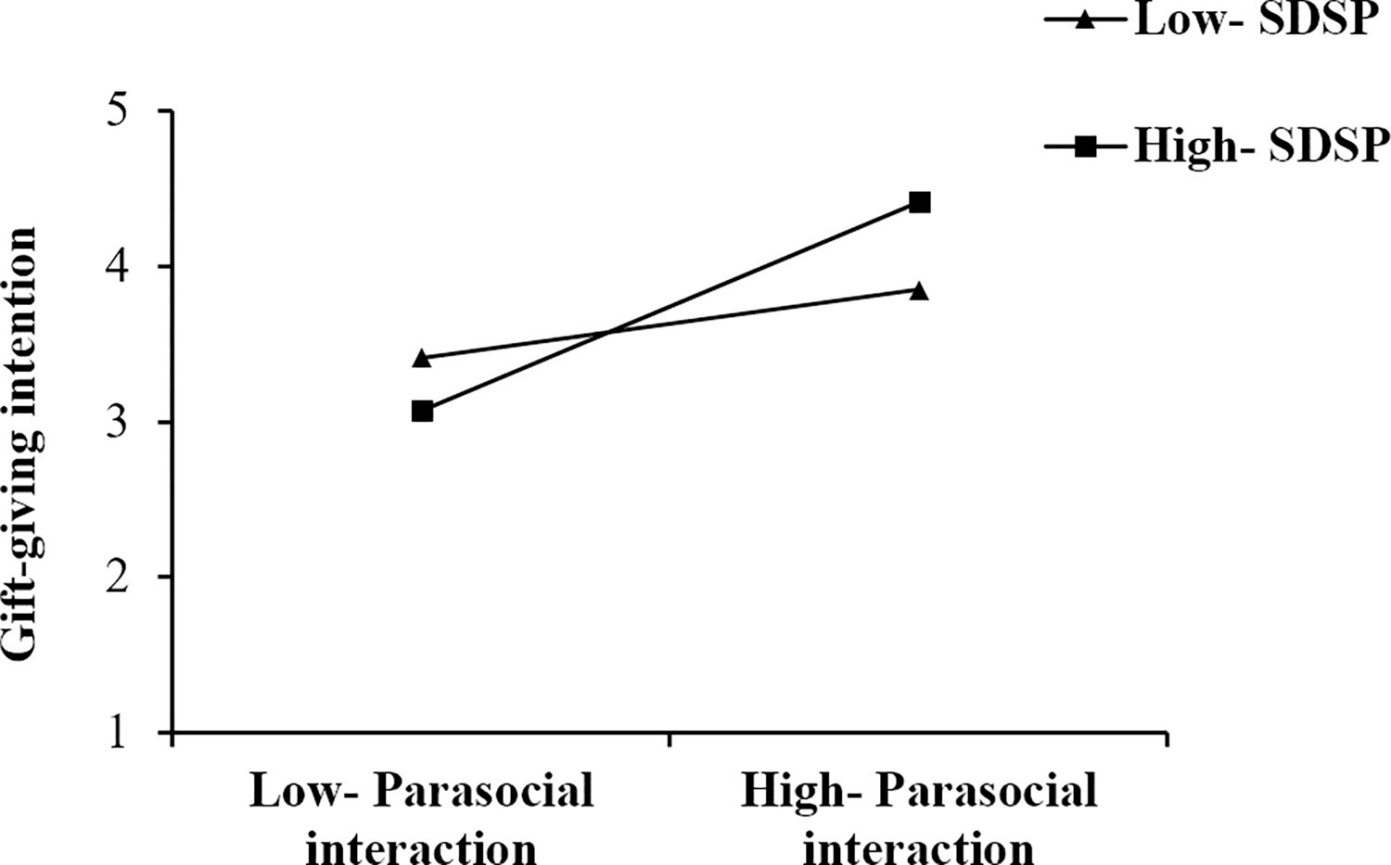
The moderating effect of SDSP on the relationship between PSI and INT.

**Fig 6:**
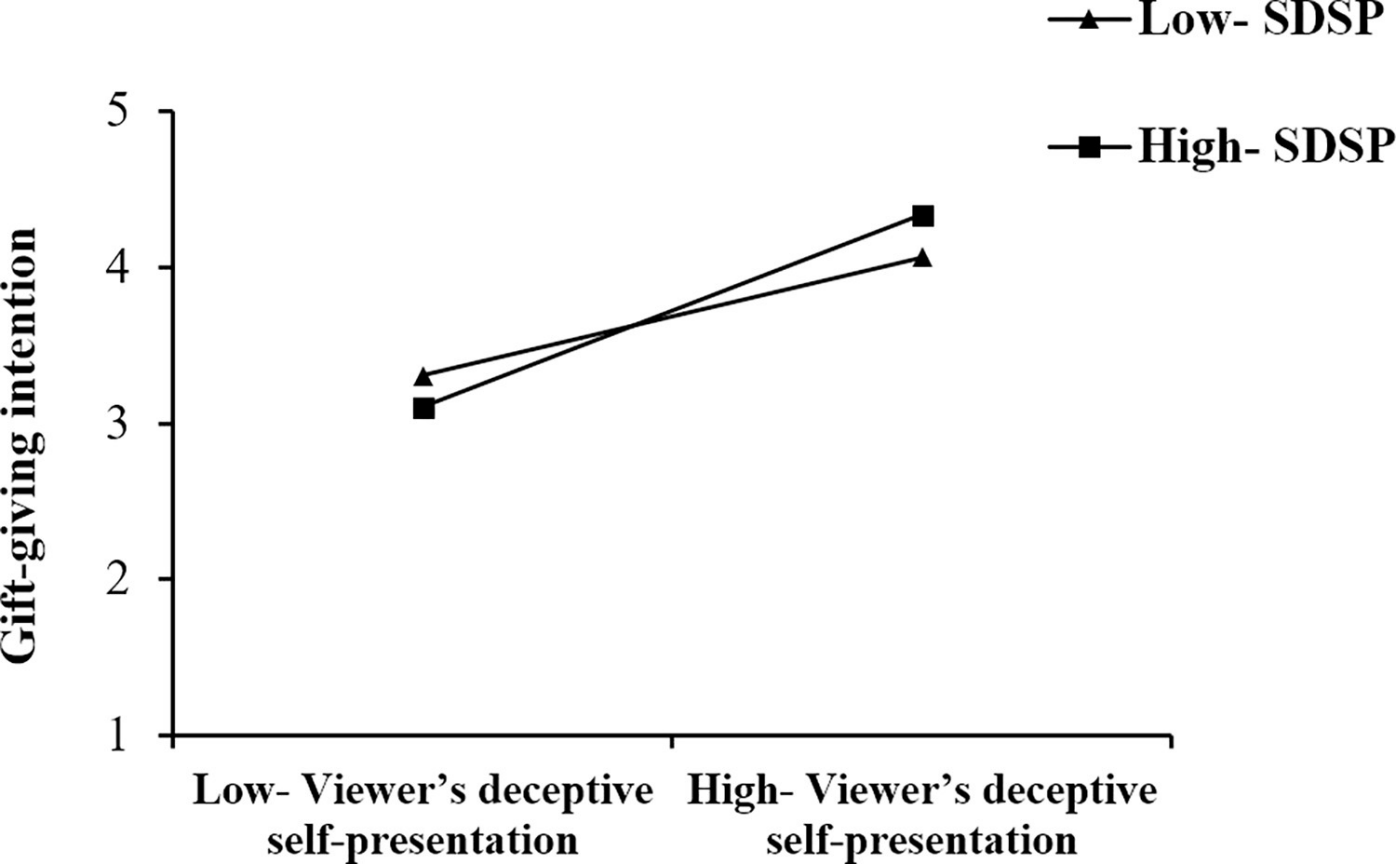
The moderating effect of SDSP on the relationship between VDSP and INT.

The moderation results are reported in Table 6. According to Table 6, This study found that the positive relationship of streamer attractiveness (ATR) and gift-giving intention (INT) is stronger when streamer’s deceptive self-presentation is high (Mean +1SD); the positive relationship of streamer expertise (EXP) and gift-giving intention (INT) is stronger when streamer’s deceptive self-presentation is high (Mean +1SD); the positive relationship of parasocial interaction (PSI) and gift-giving intention (INT) is stronger when streamer’s deceptive self-presentation is high (Mean +1SD); the positive relationship of viewer’s deceptive self-presentation (VDSP) and gift-giving intention (INT) is stronger when streamer’s deceptive self-presentation is high (Mean +1SD). To further examine the moderating effects, simple slope plots were constructed, as shown in Figs 3–6. Thus, hypotheses 5–8 are supported.

**Table 6:** Results of moderating effects.

Independent variable	SDSP	Effect	se	t	p	LLCI	ULCI
**ATR**	Mean-1SD	0.265	0.074	3.564	0.000	0.119	0.411
	Mean+1SD	0.583	0.062	9.408	0.000	0.461	0.705
**EXP**	Mean-1SD	0.267	0.073	3.632	0.000	0.122	0.411
	Mean+1SD	0.562	0.065	8.685	0.000	0.435	0.690
**PSI**	Mean-1SD	0.259	0.082	3.169	0.002	0.098	0.420
	Mean+1SD	0.790	0.074	10.748	0.000	0.646	0.935
**VDSP**	Mean-1SD	0.360	0.067	5.407	0.000	0.229	0.490
	Mean+1SD	0.586	0.063	9.345	0.000	0.462	0.709

Note: ATR, streamer attractiveness; EXP, expertise; PSI, parasocial interaction; VDSP, viewer’s deceptive self-presentation; SDSP, streamer’s deceptive self-presentation

## Discussion

### Discussion of key findings

This study builds an influencing mechanism model of the gift-giving intention of pan-entertainment live stream viewers based on social exchange theory, considering the streamer’s attractiveness, expertise, parasocial interaction, the viewer’s deceptive self-presentation, and the streamer’s deceptive self-presentation. This study was motivated by the need for a theoretical explanation of the factors that influence viewers’ gift-giving intentions and how a streamer’s deceptive self-presentation affects factors influencing gift-giving intentions. The empirical results support the hypothesized research model and are applicable to live streaming platforms used by Chinese consumers.

The following presents the findings resulted from testing hypotheses and empirical analysis. First, the attractiveness and expertise of the streamer, the degree of parasocial interaction, and the viewer’s deceptive self-presentation significantly positively affect the viewer’s gift-giving intention. These findings agree with empirical findings of [11, 22, 51, 65] that highlight the crucial role of attractiveness, expertise, parasocial interaction, and viewer’s deceptive self-presentation factors in shaping viewer behavior. Building on these studies, this study further confirms the role of these factors in the pan-entertainment livestreaming context where viewer spend money on forming close connections with streamers rather than specific products.

Second, this paper verified that the streamer’s deceptive self-presentation moderates the relationship between attractiveness, expertise, parasocial interaction, the viewer’s deceptive self-presentation factors, and the viewer’s consumer intention. This study determined that a high streamer’s self-presentation could enhance the positive impact of various factors on gift-giving behavior. This outcome broadens the scope of research on live gift-giving intentions [2, 72] by elucidating how the streamer’s self-presentation can amplify the effects of other influencing factors.

### Theoretical implications

The study of pan-entertainment livestreaming is becoming a topic of increasing interest as the digital economy grows. This research investigated the roles of streamer attractiveness, streamer expertise, parasocial interaction, viewers’ deceptive self-presentation and streamers’ deceptive self-presentation. This research contributes to the existing literature on pan-entertainment live streaming in three main ways:

First, this study extends the literature on live streaming by exploring the antecedents of viewers’ gift-giving intention. Prior research has focused on the motivations of consumers in live streaming or social media based on signal theory, technological-related motivations, or flow theory, including purchasing, interacting, and impulsive buying behavior [11, 22, 60, 65, 73]. These studies mostly take a viewer perspective to examine gift-giving intentions during pan-entertainment live streaming. This research adopts the social exchange theory to examine the factors affecting viewers’ intentions to give gifts to streamers in pan-entertainment live streaming. We extend the social exchange theory by examining the role viewer’s deceptive self-presentation in the context of pan-entertainment live streaming platforms. This incorporation provides insights into the intricacies of social exchange that takes place within pan-entertainment livestreaming rooms. This variable also generates implications regarding how viewers on digital platforms redesign their persona to selectively reveal specific aspects of their identity and personality to elicit specific reactions from viewers. This incorporation also sheds light on the motivations and expectations that viewers have on streamers, as well as the complexity inherent in online interactions.

Second, this study also identified the effect of streamers’ deceptive self-presentation on viewers’ gift-giving intentions. According to [58]’s suggestions, the context in social media spaces encompasses much more than the initially conceptualized physical and temporal environment; the majority of available research on online self-presentation is qualitative, which provides a detailed description of the reality for many social media users but does not imply effects for the majority of people; additional quantitative studies will provide more conclusive evidence in support of or opposition to the hypothesis. This study highlighted the impact of streamers’ deceptive self-presentation as a moderator on viewers’ gift-giving intentions on pan-entertainment live streaming.

Third, the social exchange theory well explains the overall model framework. 1) The viewers recognize the attractiveness and expertise of the streamer, leading them to believe that they have a unique sense of style or that the streamer’s characteristics match their own. Parasocial interaction is a crucial atmosphere stimulus that strongly affects the emotional state of viewers [65]. Parasocial interaction can make viewers feel as though they are in face-to-face contact with the streamer, as opposed to inter-acting with them online. 2) Moreover, parasocial interaction can give the viewer the motivation to improve their social presence in the eyes of the streamers. 3) The viewer’s self-deceptive presentation was previously studied in the field of online dating apps [33]. In the current study, the viewer can make a good impression on other viewers and even streamers through self-deceptive presentation and by highlighting their status, encouraging them to be willing to give gifts, as only continuous giving can highlight their high social status. 4) In addition, the streamer’s deceptive self-presentation catalyzes the process of various factors influencing gift-giving intentions. Different from the previous research findings of [49] on the purchasing behavior of live streaming e-commerce, the more the streamer’s personality evokes the viewer’s idealized image of the streamer, the greater the viewer’s propensity to give gifts.

### Practical implications

The findings of this study have several practical implications for live streamers and pan-entertainment live streaming platforms. First, the level of social presence consistency of streamers and viewer groups is an important antecedent for viewers’ gift-giving intentions. Our research presents a novel method for practitioners. Streaming platform operators can strengthen the ties between viewers and streamers to encourage more viewers to give virtual gifts.

Second, platform managers and operators should carefully consider how to cultivate viewer loyalty based on streamer and viewer groups. During the design and operation phases of a platform, certain measures can be taken to expand the scope of parasocial interactions. On the one hand, viewers can deepen their affection for the streamer by engaging in numerous parasocial interactions. The experience of par-asocial interaction can be improved by providing features that facilitate communication between streamers and viewers. Alternatively, by enhancing the expertise of pan-entertainment streamers and achieving heterogeneity within the streamer group, differentiated live streaming content can increase the viewer’s gift-giving intentions.

Third, this study concludes that the deceptive self-presentation of streamers can increase the viewer’s intention to give gifts. To ensure the fairness of the live streaming environment, the platform should implement relevant measures to limit the deceptive self-presentation of streamers, such as verifying personal real photos and identity information in the background, to prevent viewer gift-giving behavior resulting from the streamer’s misleading behavior.

### Limitations and future research

This study has several limitations. First, this study validated research model using self-reported cross-sectional data. Although we conducted a test for common method bias, the results also confirmed that there was no serious problem with common method bias. To test the stability of model variables, it is recommended that future studies employ cross-lagged methods or other techniques. Second, because of the development of AI technology, virtual streamers have emerged as live streaming formats in the pan-entertainment live streaming industry. Future research can concentrate on the application of AI technology to the field of pan-entertainment live streaming to conduct in-depth studies.

## Supporting information

S1 Appendix (DOCX)

S1 Dataset (XLSX)
