# Supplementary material for: Gift-giving intentions in pan-entertainment live streaming: Based on social exchange theory
Source: PLoS One. 2024 Jan 17;19(1):e0296908. doi: 10.1371/journal.pone.0296908 (PMC10793923; doi:10.1371/journal.pone.0296908)
Supplement: S1 Appendix — (DOCX) [file pone.0296908.s001.docx]

**Appendix**

**Measurements**

**Streamer attractiveness**

1. I think that the live stream streamer is talented.

2. I think that the streamer has an enjoyable live streaming style.

3. I think that the streamer has an interesting personality.

4. I think that the streamer has an appealing appearance.

**Expertise**

1. The streamer of the live streaming platform I watched is an expert in his/her live streaming field.

2. The professionalism of the streamer makes me feel that he/she is an expert in this field.

3. The experienced live streamer makes me feel that he/she is very profession-al.

**Parasocial interaction**

1. While I was watching, the streamer knew I paid attention to him/her.

2. While I was watching, the streamer knew that I reacted to him/her.

3. While I was watching, the streamer reacted to what I said or did.

**Viewer’s deceptive self-presentation**

1. I want to indicate my wealth.

2. I want to indicate my achievement.

3. I want to boost my image in front of others.

4. I want to gain respect.

5. I want to enhance my popularity.

6. I want to make me noticed by others.

**Streamer’s deceptive self-presentation**

1. Streamer’s self-presentation is intentionally misleading.

2. Streamer’s self-presentation is intentionally deceptive.

3. Streamer’s self-presentation is intentionally deceitful.

4. Streamer’s self-presentation is intentionally dishonest.

**Gift-giving intention**

1. Given the chance, I intend to spend money in live streaming.

2. Given the chance, I predict that I should spend money in live streaming in the future.

3. It is likely that I will spend money in live streaming in the near future.
